# Metazoan Hsp70-based protein disaggregases: emergence and mechanisms

**DOI:** 10.3389/fmolb.2015.00057

**Published:** 2015-10-09

**Authors:** Nadinath B. Nillegoda, Bernd Bukau

**Affiliations:** Center for Molecular Biology (ZMBH) of the University of Heidelberg and German Cancer Research Center (DKFZ), DKFZ-ZMBH AllianceHeidelberg, Germany

**Keywords:** Hsp70, J-protein, Hsp110, protein disaggregation, metazoan

## Abstract

Proteotoxic stresses and aging cause breakdown of cellular protein homeostasis, allowing misfolded proteins to form aggregates, which dedicated molecular machines have evolved to solubilize. In bacteria, fungi, protozoa and plants protein disaggregation involves an Hsp70•J-protein chaperone system, which loads and activates a powerful AAA+ ATPase (Hsp100) disaggregase onto protein aggregate substrates. Metazoans lack cytosolic and nuclear Hsp100 disaggregases but still eliminate protein aggregates. This longstanding puzzle of protein quality control is now resolved. Robust protein disaggregation activity recently shown for the metazoan Hsp70-based disaggregases relies instead on a crucial cooperation between two J-protein classes and interaction with the Hsp110 co-chaperone. An expanding multiplicity of Hsp70 and J-protein family members in metazoan cells facilitates different configurations of this Hsp70-based disaggregase allowing unprecedented versatility and specificity in protein disaggregation. Here we review the architecture, operation, and adaptability of the emerging metazoan disaggregation system and discuss how this evolved.

## Introduction

In healthy cells, toxicities associated with protein misfolding are countered by regulated cellular processes that sequester damaged, sticky and potentially harmful proteins to intracellular protein deposit sites (Taylor et al., 2003; Arrasate et al., 2004; Miller et al., 2015) where protein quality control machineries operate to resolve aggregates (disaggregation) (Parsell et al., 1994; Mogk et al., 1999; Tyedmers et al., 2010; Doyle et al., 2013). Accumulation of protein aggregates however is a distinguishing feature of cellular stress and aging in all organisms (Morimoto, 2008; Hipp et al., 2014) and is associated with toxicities leading to pathology (Olzscha et al., 2011; Polymenidou and Cleveland, 2012; Park et al., 2013). Aggregates hallmark a plethora of human disorders ranging from neurodegeneration to diabetes and cancers (Knowles et al., 2014; de Oliveira et al., 2015; Mukherjee et al., 2015). Persistence of protein aggregates eventually also poses a threat to the integrity of the cytoskeleton and cellular signaling (Perutz et al., 1994; Kopito, 2000; Lee et al., 2004).

The ubiquitous presence of dedicated protein disaggregation machines (disaggregases) in all cells (Winkler et al., 2012a; Doyle et al., 2013) underlines the importance of aggregate solubilization activity. Polypeptides freed from solubilizing aggregates are sorted for either refolding (Glover and Lindquist, 1998) or degradation (Ravikumar et al., 2008; Douglas et al., 2009; Ciechanover and Kwon, 2015). Proteins essential for cellular processes must be rescued and refolded via protein disaggregation activities for cell growth to resume after stress (Parsell et al., 1994; Weibezahn et al., 2004; Tessarz et al., 2008). Additionally, disaggregation and refolding activities greatly reduce resynthesis requirements (Sanchez and Lindquist, 1990; Mogk et al., 1999; Motohashi et al., 1999; Queitsch et al., 2000) which is arguably energetically favorable. Terminally damaged proteins that fail to refold are cleared from cells by proteolytic systems to prevent reaggregation and ensuing toxicities (Cuervo et al., 2004; Cohen et al., 2006). Protein disaggregation therefore, is central to the establishment of protein homeostasis and the promotion of cell survival.

## The non-metazoan Hsp100 and Hsp70•J-protein bi-chaperone disaggregation system

The ability of cells to solubilize aggregated proteins is well established in prokaryotes and in non-metazoan eukaryotes (e.g., fungi, protozoa, and plants) (Parsell et al., 1994; Hübel et al., 1997; Mogk et al., 1999; Doyle et al., 2007; Lee et al., 2007). These disaggregase machineries involve cooperation between members of the Hsp70 and the Hsp100 chaperone families (Glover and Lindquist, 1998; Goloubinoff et al., 1999; Zietkiewicz et al., 2004; Doyle and Wickner, 2009). Hsp100s are powerful AAA+ ATPases that extract trapped polypeptides from aggregates via a threading mechanism. Briefly, a hexameric Hsp100 ring with a central pore interacts with the Hsp70 system (Seyffer et al., 2012; Rosenzweig et al., 2013) to load onto protein aggregates (Winkler et al., 2012b). Concomitantly, the Hsp70 system activates the Hsp100 disaggregase for ATP-dependent substrate threading (Seyffer et al., 2012; Lee et al., 2013; Lipinska et al., 2013; Carroni et al., 2014). Protruding polypeptide termini or surface loops of trapped polypeptides are drawn into the pore to interact with flexible aromatic loop residues internal to the pore (Schlieker et al., 2004; Weibezahn et al., 2004). Unfoldase activity of Hsp70 (Sharma et al., 2011) is thought to remodel the surface of a protein aggregate through J-protein (Hsp40) controlled substrate-binding cycles, to generate these surface loops (Zietkiewicz et al., 2006). It is generally accepted that ATP hydrolysis in Hsp100 powers movement of the aromatic residues with a ratchet-type mechanism, effectively pulling the polypeptide into the pore and disentangling it from the aggregate (Lum et al., 2004; Schlieker et al., 2004; Haslberger et al., 2008). Without the cooperation of the Hsp100 disaggregase, the bacterial and yeast Hsp70 systems show very limited protein disaggregation capability (Goloubinoff et al., 1999; Diamant et al., 2000; Ben-Zvi et al., 2004; Doyle et al., 2007; Rampelt et al., 2012) inadequate for survival after severe protein aggregation stresses (Sanchez and Lindquist, 1990; Squires et al., 1991; Hong and Vierling, 2000). The Hsp100 and Hsp70•J-protein bi-chaperone disaggregation system is powerful and efficient and supports rapid response to protein misfolding stresses, minimizing cytotoxicity associated with protein aggregation (Olzscha et al., 2011; Park et al., 2013).

## The Hsp70•J-protein•Hsp110 system forms a potent metazoan disaggregase

Metazoan cells lack the core Hsp100 component of the bi-chaperone system (Doyle et al., 2013), and ambiguity in past results has made the very existence of robust protein disaggregation activity in metazoa contentious (Kampinga, 1993; Shorter, 2011; Murray et al., 2013). Recent work shows that efficient metazoan disaggregation activity requires the Hsp70 chaperone and a complex of J-proteins of two different classes (Nillegoda et al., 2015). Further cooperation with the Hsp110 co-chaperone, which acts as a nucleotide exchange factor (NEF) (Dragovic et al., 2006; Raviol et al., 2006b), boosts overall disaggregase capacity (Shorter, 2011; Rampelt et al., 2012; Nillegoda et al., 2015). This configuration provides metazoans with a potent Hsp70-based disaggregation activity that efficiently solubilizes a wide range of protein aggregates *in vitro*, comparable to the non-metazoan bi-chaperone disaggregation systems (Nillegoda et al., 2015).

**Figure 1:**
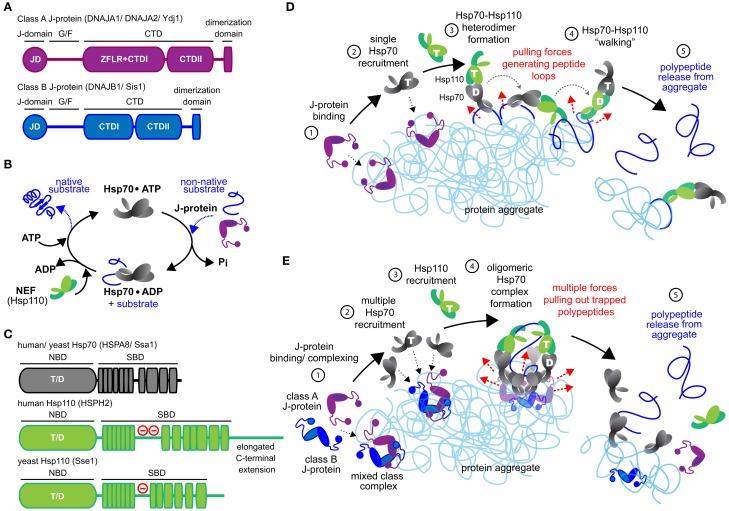
**Mechanistic models for Hsp70-based metazoan protein disaggregation. (A)** Domain organization of class A and B J-proteins (as protomers). JD designates the conserved N-terminal J-domain. G/F denotes the glycine/phenylalanine rich flexible region; ZFLR, Zinc finger-like region; CTDI and CTDII, two homologous C-terminal β-sandwich substrate-binding domains. CTDs together with ZFLR provide substrate specificity. The dimerization domain forms functional J-protein homodimers. **(B)** Hsp70•J-protein•Hsp110 functional cycle. Concomitant interaction of Hsp70 with a J-protein and substrate results in allosteric stimulation of ATP hydrolysis trapping the substrate in Hsp70. Subsequent Hsp110 mediated ADP release from Hsp70 allows ATP rebinding, which triggers substrate release to complete Hsp70 cycle. **(C)** Schematic representation of the domain organization of yeast and human Hsp110 and Hsp70. (−) in red indicates the acidic region inserted between the terminal strands of the predicted β-sheet structure. The acidic loop determines the nuclear/cytoplasmic localization of human HSPH1 (Saito et al., 2009). The extended C-terminal domain is noted in HSPH2. ATP and ADP nucleotides bound to the NBD of Hsp70 and Hsp110s are denoted as “T” and “D,” respectively. **(D)** “Clamp and walk” model for Hsp70 and Hsp110 mediated protein disaggregation. Hsp70, J-protein and Hsp110 indicated in gray, purple and green, respectively. Nucleotide state at the NBDs of Hsp70 and Hsp110 indicated by T and D. Sequential reaction steps (encircled numbers): 1, J-protein targets aggregate; 2, J-protein recruits Hsp70; 3, Hsp110 recruitment and formation of Hsp70•Hsp110 heterodimer; 4, Hsp70•Hsp110 heterodimer “walking” on aggregate by alternating scanning (ATP state) and clamping (ADP state) substrate-interaction modes that generate pulling forces (dashed red arrows) on trapped polypeptides. Pulling forces result in forming peptide loops (dark blue) that fold to native-like conformations; 5, Releasing of polypeptides from aggregate due to accumulation of native-like folding events in trapped substrates. **(E)** Metazoan “nucleation” model for efficient Hsp70-based protein disaggregation. 1, J-protein targets and nucleates on aggregate; 2, Localized, multiple Hsp70 recruitment by J-protein assemblies on aggregates; 3, Hsp110 recruitment; 4, formation of oligomeric chaperone complex containing J-protein, Hsp110 and multiple Hsp70 molecules and buildup of entropic pulling forces (dashed red arrows) leading to extraction of trapped polypeptides (dark blue). 5, Hsp110 NEF activity triggered releasing of polypeptides from aggregate.

Unraveling of aggregated proteins depends on substrate bind and release cycles of the metazoan Hsp70 in conjunction with J-protein and Hsp110 co-chaperones (Figures 1A–C). J-proteins target Hsp70 to substrates (Gamer et al., 1992; Laufen et al., 1999; Kampinga and Craig, 2010) and form the largest and the most structurally diverse chaperone family in metazoa (Cheetham and Caplan, 1998; Kampinga and Craig, 2010). Class A and B J-proteins (Figure 1A) contain conserved N-terminal J-domains (JDs) that interact with Hsp70 (Tsai and Douglas, 1996; Suh et al., 1999) and C-terminal domains (CTDs) involved in substrate binding (Lee et al., 2002; Li et al., 2003). Class A proteins contain a further zinc-finger-like region (ZFLR), also contributing to substrate recognition/binding (Lu and Cyr, 1998).

Concomitant interaction of the Hsp70 with a J-protein and substrate provides allosteric stimulation for ATP hydrolysis in Hsp70 (Bukau and Horwich, 1998; Laufen et al., 1999; Mayer and Bukau, 2005). This dual trigger traps aggregate substrate in the Hsp70 substrate-binding pocket. Substrate dissociation re-starts the Hsp70 chaperone cycle and requires release of the hydrolysis products, ADP+P_*i*_, followed by binding of a new ATP molecule to Hsp70 (Figure 1B). ADP release from Hsp70 is triggered preferentially by the Hsp110 NEF during protein disaggregation (Rampelt et al., 2012). Here, we outline the newly defined architecture and function of efficient metazoan Hsp70•J-protein•Hsp110 disaggregation machineries and briefly discuss ensuing physiological implications and evolutionary considerations.

## Hsp110 and metazoan protein disaggregation

Hsp110 chaperones are a distinct eukaryotic branch of the conserved Hsp70 superfamily (Lee-Yoon et al., 1995; Yasuda et al., 1995; Easton et al., 2000) and share the Hsp70 bipartite domain architecture: an N-terminal nucleotide-binding domain (NBD) linked to a C-terminal substrate-binding domain (SBD) (Figure 1C). Hsp110 isoforms (three in humans, Hsp105α/HSPH1, Apg-2/HSPH2, and Apg-1/HSPH3) form one of the three distinct classes of NEFs (along with Bag-type and HspBP1-type), which interact with Hsp70 molecules (Dragovic et al., 2006; Raviol et al., 2006b; Shaner et al., 2006). *In vitro*, all three cytosolic human Hsp110-type NEFs support protein disaggregation equally (Rampelt et al., 2012). However, Hsp105α knockout mouse cells show severe defects in reactivating aggregated proteins after heat stress (Yamagishi et al., 2011) despite the presence of the two other cytosolic Hsp110 members (Apg-1 and Apg-2). This apparent hierarchy among Hsp110 members *in vivo* may reflect differences in cellular localization (Saito et al., 2007) and/or tissue specific abundance (Kaneko et al., 1997; Okui et al., 2000). RNAi depletion of the single *C. elegans* cytosolic Hsp110 also shows defects in aggregate clearance after heat-stress (Rampelt et al., 2012). These *in vivo* defects most likely reflect Hsp110's role in boosting metazoan protein disaggregation identified *in vitro* (Shorter, 2011; Rampelt et al., 2012; Gao et al., 2015; Nillegoda et al., 2015). However, lack of aggregate clearance *in vivo* may also arise partly from Hsp110 involvement in holdase-type functions preventing aggregation (Ishihara et al., 2003; Yamagishi et al., 2003; Yamashita et al., 2007), and/or involvement in other protein quality control processes such as protein degradation (Heck et al., 2010; Saxena et al., 2012).

Knockdown of Hsp110, but not the Bag-1 NEF, abolishes aggregate clearance in *C. elegans* (Rampelt et al., 2012). Accordingly, substitution of Hsp110 by Bag-1 does not support efficient protein disaggregation with human Hsp70•single J-protein configuration *in vitro* (Rampelt et al., 2012; Gao et al., 2015), implying Hsp110 specialization for protein disaggregation.

The precise nature of Hsp110 specialization/function during metazoan protein disaggregation however, is currently under debate. The basic question revolves around the primary function of Hsp110 in protein disaggregation: Is Hsp110 function limited to nucleotide exchange (as a specialized NEF) or does Hsp110 function extend beyond NEF activity and act as a vital substrate-binding chaperone within the composite disaggregase machinery? The answer to this question is central to the mechanism of disaggregation.

## Evidence for Hsp110 function beyond NEF activity

Hsp110 and Bag-1 are NEFs that trigger similar structural changes in the NBD of Hsp70 inducing release of nucleotides (Sondermann et al., 2001; Andréasson et al., 2008; Schuermann et al., 2008). Why in general Bag-1 can neither substitute for Hsp110 in protein disaggregation *in vitro* nor *in vivo* is therefore puzzling. Existence of unique structural features such as an SBD (Oh et al., 1999; Goeckeler et al., 2008; Polier et al., 2010), which is absent in other types of NEFs, may support a role for Hsp110 beyond NEF activity in metazoan Hsp70-based disaggregases.

The ability of Hsp110 to directly bind aberrant protein substrates (via the SBD) is reflected in holdase activity, where Hsp110 binds to misfolding proteins and prevents thermally induced aggregation (Oh et al., 1997, 1999). Hsp110 has distinct peptide binding specificity to that of Hsp70 (Goeckeler et al., 2008; Xu et al., 2012), arising from sequence differences in the SBD (Raviol et al., 2006a). Hsp110 proteins preferentially bind aromatic residue-rich peptides, whereas canonical Hsp70s prefer aliphatic-rich peptides. Yeast Hsp110 (Sse1) exhibits reduced affinity for peptide substrate in the presence of ATP, indicating nucleotide binding induces substrate release (Xu et al., 2012). This suggests allosteric coupling between the NBD and SBD of Hsp110 proteins prompting the idea that the NEF could function as a substrate binding/unbinding Hsp70-like chaperone in protein disaggregation. However, such nucleotide dependent substrate release activity was not observed with other Sse1 specific peptide substrates (Goeckeler et al., 2008). Further, the ATP-induced peptide release activity observed by Xu and coworkers is restricted to yeast Hsp110s and is residual only, in human Hsp110 (Xu et al., 2012).

A study in fruit flies however suggests suppression of aggregation of polyQ containing proteins requires ATPase driven allosteric coupling of NBD-SBD in the fly Hsp110, since unlike wild-type fly-Hsp110, overexpression of an ATPase deficient mutant of fly-Hsp110 is unable to suppress the toxicities associated with aggregation. Suppression however, also requires co-overexpression of a J-protein (Kuo et al., 2013). The authors propose an Hsp70•J-protein-like cooperation between Hsp110 and J-proteins, beyond NEF activity. Hsp110•J-protein combinations however, are incapable of solubilizing aggregates *in vitro*. Adding Hsp70 drives solubilization (Shorter, 2011; Rampelt et al., 2012). Also, this study is *in vivo*, and therefore an Hsp70•J-protein•Hsp110 requirement is not excluded.

More compelling support for Hsp110 function beyond NEF activity comes from a study that shows human Hsp110 (Hsp105α) is an ATP-dependent foldase capable of refolding preformed misfolded polypeptides into native proteins (Mattoo et al., 2013). This study further shows a bi-directional communication linking Hsp110 and Hsp70, which allows Hsp110 to induce substrate release from Hsp70 in the absence of ATP binding. Similarly, Hsp70 induces substrate release from Hsp110. Under the conditions used, Mattoo and coworkers find optimal Hsp70•J-protein•Hsp110 disaggregase activity at a 1:1 stoichiometry for Hsp70:Hsp110 (Mattoo et al., 2013). Based on 1:1 optimal activity stoichiometry and the foldase capacity of Hsp110 (both of which activities require a J-protein to be present) these authors propose an Hsp70•Hsp110 core functional unit for metazoan disaggregases and the first mechanistic model for the metazoan Hsp70-based disaggregase.

## The Hsp70•Hsp110 “clamp and walk” model

The “*clamp and walk*” model proposed by Mattoo et al. (2013) (Figure 1D) postulates an Hsp70•Hsp110 heterodimer as the functional core unit of the Hsp70-based disaggregase. The spatial arrangement of the proposed heterodimer depicted in Figure 1D derives from the crystal structure of bovine Hsc70 NBD and yeast Hsp110 Sse1 (Schuermann et al., 2008). Hsp70 (black) and Hsp110 (green) toggle between ATP (T) and ADP (D) bound states, triggering alternately coordinated substrate binding and release for each. The ATP bound chaperone state is engaged in scanning for new proximal substrate contacts, while the other ADP-bound chaperone is anchored to the protein aggregate. Capture of a new aggregated polypeptide segment by the first molecule triggers substrate release in the anchored molecule, locally unwinding the released polypeptide segment to form an unfolded polypeptide loop. Such sequential bind-and-release events, or “walking” of the Hsp70•Hsp110 heterodimer, are predicted to constitute a power-stroke action (Sousa and Lafer, 2006), which pulls out and unfolds a series of polypeptide loops from trapped substrates on the surface of an aggregate (dark blue, Figure 1D). Polypeptide segments in these loops would then spontaneously refold to native-like conformations. Accumulation of these small refolding events along an aggregated polypeptide would promote polypeptide dissociation from the aggregate (Mattoo et al., 2013).

## Irreconcilable data

Though the “*clamp and walk*” model is attractive, it is inconsistent with accumulating and emerging data. The foldase activity of Hsp110 is debatable, as is the concerted action of Hsp70 and Hsp110 in protein disaggregation proposed by Mattoo and coworkers (Mattoo et al., 2013). Studies previous to the Mattoo report do not see any foldase activity by an Hsp110•J-protein pair (Oh et al., 1997; Yamagishi et al., 2000; Dragovic et al., 2006), although the kind of substrates used in the Mattoo analysis may have given rise to different results. In addition, mechanistic studies on yeast and human Hsp110s consistently fail to detect any hallmark features of canonical Hsp70s (Shaner et al., 2004; Raviol et al., 2006a; Liu and Hendrickson, 2007; Andréasson et al., 2008). Direct analysis shows that characteristic ATP and J-protein triggered conformational rearrangements of Hsp70 are absent in yeast and human Hsp110s (Raviol et al., 2006a; Andréasson et al., 2008; Goeckeler et al., 2008) although ATP hydrolysis occurs in Hsp110 (Raviol et al., 2006a; Goeckeler et al., 2008; Mattoo et al., 2013). Taken together, the majority of studies shows a lack of Hsp70-like allosteric coupling between NBD and SBD and foldase activity for Hsp110s (Oh et al., 1997; Yamagishi et al., 2000; Dragovic et al., 2006; Raviol et al., 2006a; Andréasson et al., 2008; Goeckeler et al., 2008). Further, a NEF activity-deficient mutant of human Hsp110, but not an ATPase deficient mutant, is defective in Hsp70•J-protein mediated protein disaggregation (Rampelt et al., 2012). This strongly suggests the primary function of Hsp110 in protein disaggregation is nucleotide exchange and not an activity requiring ATP-dependent structural rearrangements in Hsp110.

In general, NEFs act at substoichiometric levels to Hsp70 to avoid futile nucleotide exchange cycles in Hsp70 leading to inhibitory effects during non-metazoan protein disaggregation and/or refolding assays *in vitro* (Goloubinoff et al., 1999; Yamagishi et al., 2000; Zietkiewicz et al., 2006; Genest et al., 2011). Human Hsp110 displays characteristics of a typical NEF during metazoan protein disaggregation/refolding and works optimally at substoichiometric levels relative to Hsp70. Accordingly, higher Hsp110 to Hsp70 ratios inhibit protein solubilization by the human Hsp70-based disaggregation system (Rampelt et al., 2012; Gao et al., 2015; Nillegoda et al., 2015). However, under the conditions Mattoo and colleagues use, highest protein disaggregation activity is found at equimolar concentrations of Hsp70 and Hsp110 (Mattoo et al., 2013). This contradiction in Hsp70 to Hsp110 stoichiometry perhaps arises from differences in experimental conditions. The rationale for an Hsp70•Hsp110 heterodimer based disaggregase model however, depends heavily on this observation. Furthermore, structurally, the elongated C-terminal extension (Figure 1C) unique to human Hsp110s and important for substrate binding (shown for holdase activity) (Oh et al., 1999; Raviol et al., 2006a) is superfluous for protein disaggregation. The yeast Sse1 NEF which has a stunted C-terminal extension functionally dispensable *in vivo* (Shaner et al., 2004; Liu and Hendrickson, 2007) and *in vitro* for NEF activity (Andréasson et al., 2008), is capable of fully substituting for the human Hsp110 during protein disaggregation (Rampelt et al., 2012). This strongly suggests, but does not formally show, that substrate-binding features of human Hsp110 are dispensable for disaggregation. Altogether, these observations further consolidate a primarily nucleotide exchange function for metazoan Hsp110 in protein disaggregation. Finally and most tellingly, Hsp110 is not strictly essential for activity in some disaggregase configurations (Nillegoda et al., 2015), though not others (Gao et al., 2015). The *in vitro* activity on amorphous aggregates by human Hsp70-based disaggregases containing heterocomplexed J-proteins is ~33% less efficient without Hsp110 (Nillegoda et al., 2015). This is reminiscent of the yeast bi-chaperone-based disaggregase system where Hsp110 acts as a NEF, which boosts, but is dispensable for, disaggregation (Glover and Lindquist, 1998; Rampelt et al., 2012).

The “*clamp and walk*” model is unclear as to how J-proteins, an essential component of metazoan disaggregase machinery (Nillegoda et al., 2015), participate in the “walking” dynamics of the proposed Hsp70•Hsp110 heterodimer on the aggregate (Figure 1D, beyond step 1). Cumulatively, these points suggest a central role for an Hsp70•Hsp110 heterodimer in metazoan protein disaggregation is unlikely, and point instead to a central involvement of J-proteins.

In short, both the analytical discrepancies outlined regarding NEF independent function and the latest developments in Hsp70-based disaggregase biology involving J-protein requirements (Nillegoda et al., 2015) suggest the primary role of Hsp110 in disaggregation is nucleotide exchange. These considerations argue against a central architectural role for Hsp110 as a key substrate-binding chaperone in the Hsp70-based disaggregases.

## Metazoan NEF specialization in protein disaggregation

One explanation for the specialization of Hsp110 in protein disaggregation may lie in the kinetics of NEF driven Hsp70 cycling through ATP/ADP states. A further consideration is the overall architecture of the assembled Hsp70 disaggregase, which may limit steric accessibility of one NEF over another in some configurations. Hsp110 and Bag1 NEFs utilize a similar mechanism to induce nucleotide release from Hsp70, but have discrete binding interfaces on the Hsp70 NBD (Sondermann et al., 2001; Andréasson et al., 2008). The assembled disaggregase core architecture (J-proteins and Hsp70) probably favors accessibility of one binding interface (Hsp110) over the other (Bag1), depending on the specific J-protein combinations incorporated into the core architecture. This accounts well for the inability of the Bag-1 NEF to promote efficient nucleotide exchange in Hsp70-based protein disaggregation in some combinations but not others. Hsp70•DNAJB1•Bag-1 combinations show poor disaggregation activity compared with Hsp70•DNAJB1•Hsp110 combination. In contrast, Hsp70•DNAJA2 combinations, which specifically target smaller aggregates (Nillegoda et al., 2015), function equally well with Hsp110 and Bag-1 *in vitro* (Rampelt et al., 2012). Bag-1 displays similarly differential cooperative efficacy in refolding proteins, with Hsp70•DNAJA2 providing highest activity (Terada and Mori, 2000). Although not directly shown, these observations are entirely consistent with steric exclusion due to architectural constraints leading to lack of function, and suggest Hsp110 has evolved to provide a specialized NEF used by all Hsp70-based disaggregases.

On balance, we predict that Hsp110 co-chaperones play a dual role in protein disaggregation. Hsp110 primarily provides NEF activity facilitating efficient substrate release from Hsp70 molecules, which resets the disaggregase machine for another round of polypeptide extraction. The contribution of Hsp110 NEF activity to protein disaggregation is dispensable and varies with J-protein class (Rampelt et al., 2012), J-protein class cooperation (Nillegoda et al., 2015), and substrate-type (Gao et al., 2015; Nillegoda et al., 2015). Hsp110 however may also perform an extra, but non-essential holdase function in disaggregation by interacting directly with polypeptides during extraction from the aggregate, as invoked by the earlier “*clamp and walk*” model (see Figure 1E for a newly proposed model for Hsp70-based disaggregases).

## The metazoan “nucleation” model for efficient Hsp70-based protein disaggregation

A different mechanism for metazoan Hsp70-based disaggregation incorporates the latest data (Figure 1E) and resolves the analytical discrepancies outlined. This mechanism involves initial formation of oligomeric, higher order chaperone structures containing multiple Hsp70 molecules on the aggregate surface. Clustered binding of Hsp70 molecules, potentially to the same trapped substrate polypeptide, will increase the extracting force on the polypeptide due to decreasing entropy (De Los Rios et al., 2006; Goloubinoff and De Los Rios, 2007), facilitating local disaggregation. Repulsive forces generated by steric exclusion of bulky clustered Hsp70 molecules are also proposed to disrupt strong peptide-peptide interactions (Kellner et al., 2014). Together, such forces are thought to help release trapped polypeptides from aggregates. Unfolding is a prerequisite for subsequent correct protein refolding of extracted polypeptides. Unlike non-metazoan Hsp100 AAA+ ATPases where extracting polypeptides are unfolded by threading through a molecular tunnel (Weibezahn et al., 2004; Hinnerwisch et al., 2005; Haslberger et al., 2008; Doyle et al., 2012), the metazoan Hsp70-based disaggregase probably relies instead on the unfoldase power of Hsp70 chaperones to directly unravel the disaggregating polypeptides (Sharma et al., 2011). The multi-component disaggregase complex may also form a channel-like or cavity-like structure to stabilize the disaggregating, unfolded polypeptide (Figure 1E, step 4). However, how are multiple Hsp70 molecules efficiently attracted to one site on the surface of an aggregate? This is the crucial first step for this model.

## A central role for J-proteins in disaggregase structure

Recent work reveals the formation of transient heterocomplexes between class A and class B homodimer J-proteins via intermolecular JD•CTD interactions. These mixed class J-protein complexes formed on the surface of amorphous aggregates boost the efficacy of metazoan Hsp70-based disaggregases (Nillegoda et al., 2015). Canonical J-protein homodimers present two J-domains for potential interaction with two independent Hsp70 molecules (Morgner et al., 2015). On this basis, a minimal mixed-class dimer-dimer J-protein complex would present four J-domains and could therefore recruit up to four Hsp70 molecules after binding to an aggregate (Figure 1E, step 2). Conglomeration of Hsp70 molecules would further increase if recruited Hsp70 molecules themselves further formed homodimers, as recently seen in bacteria (Malinverni et al., 2015; Sarbeng et al., 2015). J-protein nucleation on the surface of protein aggregates therefore would provide a foundation upon which multiple Hsp70 molecules are recruited to form oligomeric Hsp70-based efficient disaggregation machines. The precise basis for J-proteins nucleation on aggregates has not been defined but presumably J-proteins nucleate where looped out polypeptide stretches are available for binding.

## Summarizing the support for the two models

The crucial difference between the two models lies in the molecular architecture of the core disaggregase, which dictates mechanism of aggregate solubilization. In the earlier model an Hsp70•Hsp110 heterodimer core enables a ratcheted bind-and-release of aggregate substrate, in a “walking” disaggregation action to create successive disaggregated domains on a polypeptide, eventually leading to full disaggregation. This presumes ATP-hydrolysis coordinates substrate capture and release by Hsp70, which is well established, but also for Hsp110, which is experimentally unsupported. Further, recent data show Hsp110 is not strictly essential in some metazoan disaggregase configurations. J-proteins on the other hand, are indispensable. The clamp and walk mechanism, based on an Hsp70•Hsp110 core architecture, strictly requires Hsp110, and makes no provision for J-protein function other than the initial targeting of Hsp70 or Hsp110 to the aggregate. Together, these points make an Hsp70•Hsp110 heterodimer architecture and the ensuing ratchet mechanism less plausible.

In contrast, the new model requires initial nucleation by J-proteins for Hsp70-based disaggregation to proceed. In this model J-proteins target amorphous aggregate surfaces, recruiting multiple Hsp70 molecules via established interaction interfaces, to foci on the aggregate, nucleating higher order Hsp70-J-protein core disaggregase structures. The NEF activity of Hsp110 is beneficial and often essential for enhancing disaggregation function, but for some disaggregase configurations dispensable. There is also evolutionary precedence suggesting a bacterial J-protein-mediated Hsp70 clustering mechanism driving Hsp100 dependent disaggregation (Seyffer et al., 2012). In the metazoan context, J-protein nucleation on aggregate surfaces is therefore very plausible.

## A J-protein gearbox regulates metazoan protein disaggregation efficacy

Both efficacy and substrate (protein aggregate) specificity of Hsp70-based disaggregases are determined by J-proteins. Hsp70-based disaggregases containing class A vs. class B J-proteins specifically target different amorphous aggregates. Human Hsp70 and Hsp110 combined with class A J-protein (DNAJA1, DNAJA2) targets only small aggregates (Mattoo et al., 2013; Nillegoda et al., 2015). In contrast, the Hsp70•class B J-protein (DNAJB1)•Hsp110 system solubilizes only large aggregates (Nillegoda et al., 2015). This explains the superior disaggregation activity of the Hsp70•DNAJB1•Hsp110 combination in previous work which used substrates consisting predominantly of large aggregates (i.e., aggregated luciferase formed under high luciferase concentration) (Rampelt et al., 2012). Selection is based on aggregate size/structure rather than substrate type, possibly arising from differences in class A vs. B J-protein mode of binding (Terada and Oike, 2010) and/or peptide binding characteristics (Fan et al., 2004). Unlike single class J-proteins, mixed class J-protein complexes provide broad substrate specificity, allowing Hsp70-based disaggregases to target aggregates over a wide size range (Nillegoda et al., 2015). This is most likely due to combined presence of different substrate binding CTDs in the complex. Different aggregate types (amorphous vs. amyloid) are resolved by markedly different configurations of Hsp70-based disaggregases. For example, the Hsp70•DNAJB1•Hsp110 single J-protein configuration, which specifically targets large amorphous aggregates is also sufficient for efficient disintegration of α-synuclein amyloid fibrils and does not require mixed class J-protein complexing (Gao et al., 2015).

**Figure 2:**
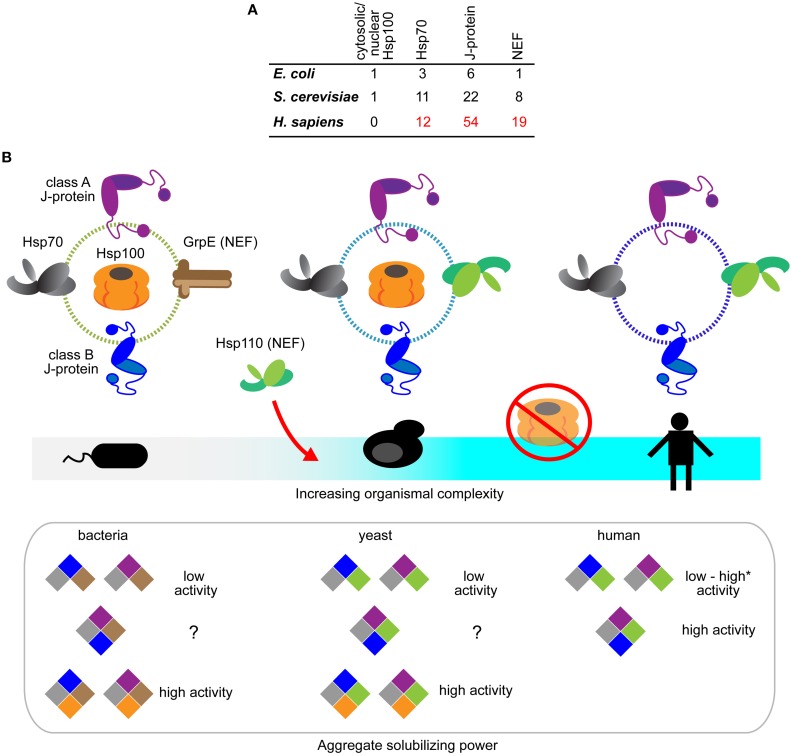
**Evolution of the Hsp70-based protein disaggregases. (A)** Tabulation of Hsp100, Hsp70, J-protein and nucleotide exchange factors in prokaryotes (*E. coli*), non-metazoan eukaryotes (*S. cerevisiae*), and metazoa (*H. sapiens*). The listings include experimentally established isoforms of the respective chaperones and co-chaperones (Genevaux et al., 2001; Lu et al., 2006; Saito et al., 2009; Kampinga and Craig, 2010). **(B)** Schematic diagram depicting the emergence of Hsp70-based protein disaggregases and loss of Hsp100 and Hsp70 bi-chaperone machines during evolution. Hsp100, GrpE (bacterial NEF), Hsp70, J-proteins and Hsp110 indicated in orange, brown, gray, purple (class A J-protein), blue (class B J-protein) and green, respectively. Hsp110 NEFs only appear in eukaryotes. Hsp100 is absent in metazoan cytosol and nucleus. Aggregate solubilization power of bacterial, yeast and human disaggregation systems indicated bellow. Color-coding depicts different components of the disaggregation systems. ^*^Denotes the high protein disaggregation activity by Hsp70•JB1•Hsp110 configuration that rapidly disassemble α-synuclein fibrils (Gao et al., 2015).

Overall, it is clear that during protein disaggregation, J-proteins can function both independently in a class-dependent manner, and as mixed-class complexes with markedly distinct properties, dependent on specific constituent J-proteins. Humans have over 50 members in the J-protein family (Figure 2A) (Kampinga and Craig, 2010), as do other metazoans like *C. elegans* (~30 members) (Yook et al., 2012). A wide range of complexed J-protein combinations is therefore available to metazoa, essentially providing a metazoan gearbox for fine-tuning target selectivity and efficacy of protein disaggregation.

## The emergence of Hsp70-based protein disaggregases during evolution

Gene losses occur in all major lineage transitions of life. Such losses are usually reflected as deficiencies in specific biological activities (Danchin et al., 2006). The abrupt loss of cytosolic/nuclear Hsp100 class members in the transition to metazoa has no immediately obvious basis, since protein disaggregation activity is preserved and is essential in the metazoa.

Loss of Hsp100 during metazoan evolution coincides with gain-of-disaggregation function in Hsp70 machines during metazoan evolution (Figure 2B). Three major changes in cellular protein quality control could account for reduction of the disaggregation machine from an Hsp100-Hsp70 dual system to the single Hsp70 system: (1) The appearance of vacuolar/lysosomal-based autophagic protein degradation in eukaryotes diversifies and augments mechanisms of aggregate clearance in metazoa (Lu et al., 2014; Rogov et al., 2014). Presence of an alternative pathway could reduce selection pressure for the relatively energy-expensive Hsp100. (2) Habitat wise, free living bacteria, fungi and plants are exposed to constantly changing harsh environmental stresses, unlike metazoans, and rely heavily on Hsp100-based disaggregases for survival after extreme heat stress (Sanchez and Lindquist, 1990; Squires et al., 1991; Hong and Vierling, 2000). Hsp100 however is dispensable for central biological processes (Hong and Vierling, 2001) and under unstressed growth conditions is actually detrimental to fitness (Escusa-Toret et al., 2013). The fitness cost associated with maintaining a powerful Hsp100-based disaggregase system therefore, may have driven better stress-buffering in metazoan cells (Durieux et al., 2011; Gidalevitz et al., 2011; Van Oosten-Hawle et al., 2013) and loss of Hsp100. (3) The emergence of enhanced disaggregation versatility, via J-protein and NEF configurations providing a highly tunable Hsp70-based protein disaggregation system, may have also contributed to loss of Hsp100. A substrate-tailored versatile disaggregation system is better suited to the needs of multicellular organisms than the potent, but inflexible and less specialized Hsp100-based bi-chaperone disaggregase system found in non-metazoan life-forms.

Metazoan Hsp70, particularly the constitutive Hsc70 (HSPA8) also harbor critical evolutionary changes that support protein disaggregation. Appearance of Hsp110 in non-metazoan eukaryotes may have triggered concomitant development of accessorizing features of the partner protein Hsp70. Yeast Sse1 boosts the activity of human Hsp70•J-protein (HSPA8•DNAJB1) disaggregation system, but is unable to do so to the same level for yeast counterparts (Ssa1•Ydj1 or Ssa1•Sis1) (Rampelt et al., 2012). This points clearly to specialization of metazoan Hsc70 in protein disaggregation and this remains to be dissected. What also remains unclear is the evolution of mixed class J-protein complexing in protein disaggregation, especially since both class A and class B J-proteins exist in non-metazoans (Figure 2B).

## Concluding remarks

In metazoans, the expanded number of Hsp70, J-protein and NEF class members enables greater flexibility of disaggregase machinery configuration, suggesting a natural selection in favor of versatility of function. However, increased system diversification, versatility and components also increases the scope for defects arising in protein quality control processes, with the potential to translate into disease.

The substrate spectrum of the metazoan Hsp70-based disaggregase is currently poorly understood *in vivo*. It is of particular interest to examine how disease-linked amyloid-type aggregates that form stable fibrils can be disassembled by Hsp70-based disaggregases. Components of the human Hsp70-based disaggregase have been isolated from a variety of amyloid-type aggregates (Olzscha et al., 2011; Kirstein-Miles et al., 2013; Song et al., 2013) indicating that Hsp70 machinery may play a role in amyloid related neuropathies. A recent *in vitro* study now shows a specific architecture of the Hsp70•Hsp110•J-protein configuration rapidly disassembles α-synuclein fibrils, via a fibril-specific mechanism, involving both fragmentation and depolymerization (Gao et al., 2015). This is particularly exciting, as the timeframe of disassembly is physiologically relevant. The full physiological impact, interplay and function of metazoan disaggregase machines *in vivo* however, remains largely unexplored and the most immediate challenge is to dissect the molecular composition, dynamics, and regulation of the basic disaggregation process in human cells.

### Conflict of interest statement

The authors declare that the research was conducted in the absence of any commercial or financial relationships that could be construed as a potential conflict of interest.
